# Environmental effects shape the maternal transfer of carotenoids and vitamin E to the yolk

**DOI:** 10.1186/1742-9994-9-17

**Published:** 2012-08-09

**Authors:** Wendt Müller, Jonas Vergauwen, Marcel Eens, Jonathan D Blount

**Affiliations:** 1 Department of Biology-Ethology, University of Antwerp, 2610, Wilrijk, Belgium; 2 Centre for Ecology and Conservation, University of Exeter, Cornwall, TR10 9EZ, UK

**Keywords:** Antioxidants, Environmental effect, Food manipulation, Maternal effect, Yolk hormones, Testosterone

## Abstract

**Introduction:**

Maternal effects occur when the phenotype of the offspring is influenced by the phenotype of the mother, which in turn depends on her heritable state as well as on influences from the current and past environmental conditions. All of these pathways may, therefore, form significant sources of variation in maternal effects. Here, we focused on the maternal transfer of carotenoids and vitamin E to the egg yolk, using canaries as a model species. Maternal yolk carotenoids and vitamin E are known to generate significant phenotypic variation in offspring, representing examples of maternal effects. We studied the intra-individual consistency in deposition patterns across two years and the mother-daughter resemblance across two generations in order to estimate the level of heritable variation. The effects of the current environmental conditions were studied via a food supplementation experiment, while the consequences of past environmental conditions were estimated on the basis of the early growth trajectories.

**Results:**

There was a significant effect of the current environmental conditions on the yolk carotenoid and vitamin E deposition, but this effect varied between antioxidant components. The deposition of yolk carotenoids and vitamin E were linked to the process of yolk formation. Past environmental conditions did not contribute to the variation in yolk carotenoid and vitamin E levels nor did we find significant heritable variation.

**Conclusions:**

The transfer of carotenoids or vitamin E may be an example where current environmental variation is largely passed from the mother to the offspring, despite the numerous intermediate physiological steps that are involved. Differences in the effect of the environmental conditions as experienced by the mother during laying may be due to differences in availability as well as physiological processes such as competitive exclusion or selective absorption.

## Introduction

Maternal effects occur when offspring phenotype is influenced by the phenotype of the mother, which in turn depends on the environmental conditions the mother experiences as well as on her - partly heritable - physiological state [1,2]. Maternal effects are thought to have evolved to match the phenotype of the offspring to (changes in) their environment (‘adaptive maternal effects’) [1-3]. A main focus of research has, therefore, been on identifying environmental sources of variation in maternal effects. This has been particularly well studied in birds, where egg size and composition can be considered as important maternal traits that generate significant changes in offspring phenotype [4-6]. More specifically, several recent studies have focused on a number of specific egg components such as hormones, antibodies, carotenoids and vitamins [e.g. [4,5,7,8]. In case of the latter two, there is now a large body of evidence for environmental effects modifying the maternal deposition of carotenoids and vitamins [e.g. [7-12].

However, maternal effects not only have an environmental but also a genetic component [2], both of which shape the evolutionary significance of a maternal effect. Yet even though maternal effects have been particularly well studied in birds, there is still little information available on heritable variation in maternal traits generating changes in offspring phenotype through variation in egg components. One exception to this is the maternal transfer of hormones to the yolk, for which the heritability has recently been investigated, revealing a comparatively high degree of mother-daughter resemblance [13-15] (see [6] and references therein for the heritability of egg size). However, the degree of inheritance varied with the type of hormone investigated, with high heritability estimates being found for testosterone, but not for androstenedione deposition [13,14] (but see [15]). This suggests that the heritability of a given trait may depend on the physiological mechanisms of transfer that are available to the female. It is, therefore, highly interesting to study how the level of genetic inheritance varies between different traits, in order to ultimately improve our understanding of the evolution of maternal effects in birds. At present it is obviously most rewarding to study the heritability of the maternal deposition of egg components of which the transfer to the yolk is very different from the transfer of maternal androgens - such as in case of carotenoids or vitamin E.

Maternal yolk carotenoids and vitamin E provide protection against oxidative damage and improve immune defences [16-19], representing a significant pathway for maternal effects. However, carotenoids and vitamin E cannot be synthesized de novo and must come from the environment [20], where their availability is often limited [21-25]. This is in contrast to hormones that are not physically obtained from the environment, but are produced by the female in response to environmental cues [26]. Thus, the transfer of carotenoids or vitamin E may depend much less on the (genetic) constitution of the female, but may form an example where the environmental variation passes through the maternal body/phenotype and ultimately to the offspring without much further modification [27]. But on the other hand, it has also been suggested that females may be able to regulate the deposition of carotenoids and vitamin E [10,11]. Indeed, a number of physiological processes are involved in the transfer of carotenoids and vitamins to the yolk, all of which may have a heritable basis [28,29]. The maintenance of individual variation despite super-abundant carotenoid supplementation (e.g. [30,31]) may be taken as further support for the role of regulatory physiological processes in the use and allocation of carotenoids.

Here, we investigated how genetic and environmental effects shape the maternal transfer of carotenoids and vitamin E to their progeny, using canaries (*Serinus canaria*) as a model organism. To study the degree of inheritance, we first estimated the intra-individual consistency in maternal deposition patterns across two different breeding seasons (upper-bound estimate for heritability [32]). Second, we measured the mother-daughter resemblance in yolk carotenoid and vitamin E levels, as the relative contribution of genes can ultimately only be inferred via a comparison of related individuals. This provided us with an estimate of the narrow sense heritability (defined as the ratio of the additive genetic variance to the phenotypic variance [32]). Given the important role of the environment in determining the levels of yolk carotenoids as revealed by previous studies [7-12], we also studied the contributions of two different environmental effects on yolk carotenoids and antioxidant levels. The first part investigated the effects of a diet manipulation on yolk carotenoids and vitamin E deposition. The food conditions may have an impact on their accumulation in the yolk and may also alter their covariation with other essential egg components such as yolk testosterone due to trade-offs, correlated responses or the necessity of mutual adjustment [21]. Studying the covariation among egg components may also improve our understanding of the underlying physiology. In addition, we estimated the potential consequences of variation in the growth conditions during early development for the deposition of yolk carotenoids and vitamin E at adulthood, given the evidence for trans-generational effects of developmental stress on reproductive traits at adulthood [33], and these may manifest themselves via maternal effects [14,34,35]. Interestingly, two recent studies suggested that the early developmental conditions may affect the carotenoid metabolism at adulthood: early life experiences had long-lasting effects on beak coloration in zebra finches [36] and breast plumage in great tits [37,38]. Both traits are dependent on the incorporation of carotenoids. Such changes in carotenoid metabolism are likely to have consequences for the transfer of carotenoids to the yolk. However, this has as yet not been investigated.

## Results

### Genetic effects: individual consistency and inheritance

We calculated the individual consistency in yolk carotenoids and vitamin E concentrations of the second-laid egg for 25 females of the P-generation that laid a clutch in both years of the study. The repeatability was low and non-significant for the three components studied (repeatability ± s.e.; α-tocopherol: r = 0.09 ± 0.11, F = 1.26, p = 0.28; γ-tocopherol: r = 0.02 ± 0.11, F = 1.05 p = 0.46; total carotenoids: r = −0.09 ± 0.1, F = 0.80, p = 0.71). The negative repeatability is likely to represent noise around statistical zero and should be interpreted as evidence for zero repeatability [39].

**Figure 1:**
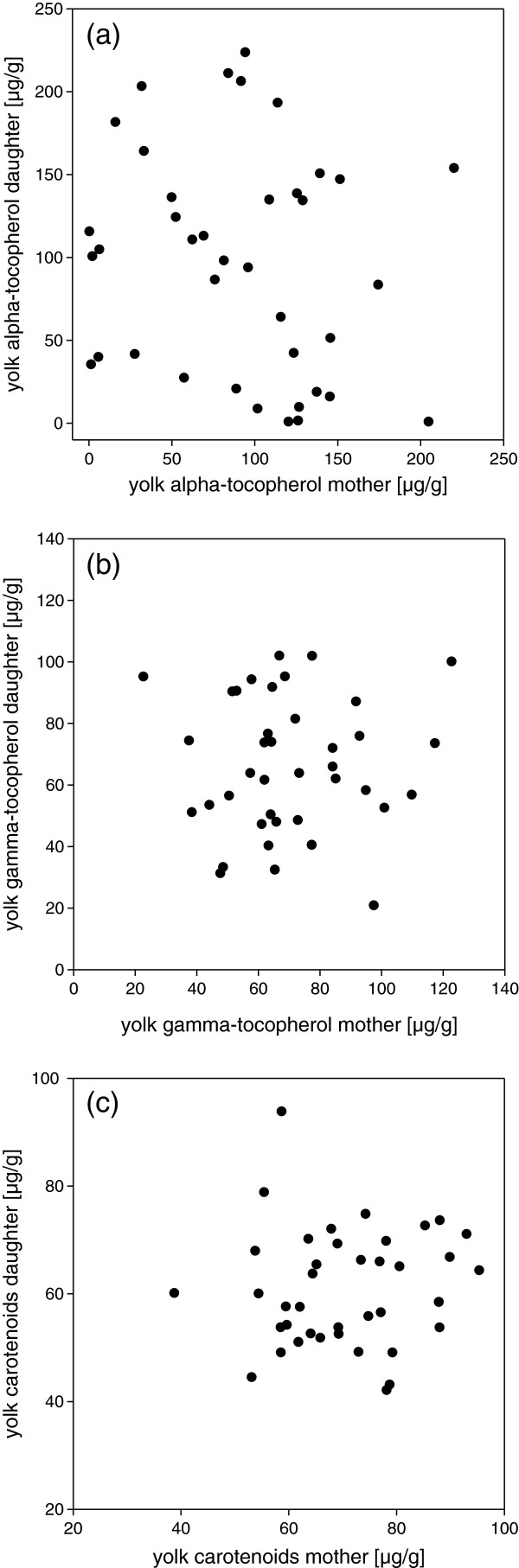
**Mother-daughter resemblance.** Comparison of the alpha-tocopherol (**a**), gamma-tocopherol (**b**) and carotenoid (**c**) concentrations in the egg yolk of mothers and daughters. In neither case was the mother-daughter resemblance statistically significant.

The heritability was studied based on 39 mother-daughter pairs and is based on the second laid egg from the clutches of 39 mothers and the 39 clutches of their respective daughters. Daughters did not significantly resemble their mothers in the yolk concentrations of α-tocopherol (b = −0.19 ± 0.19, p = 0.33; h^2^ = −0.38, 95% CI −1.14 to 0.38), γ-tocopherol (b = 0.03 ± 0.16, p = 0.86; h^2^ = 0.06, 95% CI −0.57 to 0.69) or total carotenoids (b = 0.06 ± 0.14, p = 0.68; h^2^ = 0.12, 95% CI −0.42 to 0.65) (Figure 1).

### Environmental effects: high versus low quality diet

The consequences of experimentally introduced environmental variation could be analyzed for 35 (16 high quality and 19 low quality diet females) out of the initial 44 females (see [40] for more details). High quality diet females laid heavier second eggs (F_1,33_ = 6.03, p = 0.02), which did not contain significantly more yolk (F_1,33_ = 0.01, p = 0.93), indicating that the previously described pattern also holds when considering the second-laid egg only [40].

**Figure 2:**
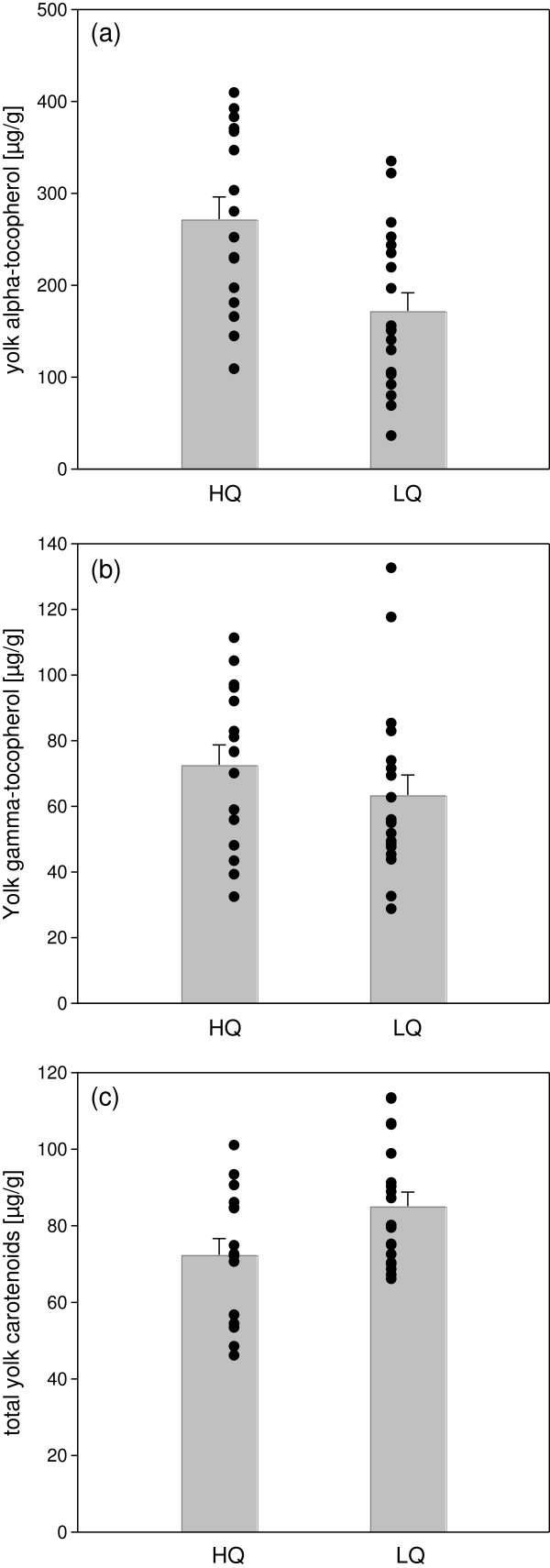
**Environmental effects acting during egg laying (food manipulation experiment).** Females on a high quality diet (HQ) deposited significantly more alpha-tocopherol in their yolk compared to females that received a low quality diet (LQ) (**a**), but there were no significant differences in the gamma-tocopherol concentrations (**b**). The egg yolk of females on a HQ diet contained significantly less carotenoids compared to the egg yolk of females on a LQ diet (**c**).

The high quality diet significantly increased the transfer of α-tocopherol to the yolk of the second laid egg, both in terms of concentration (F_1,33_ = 7.86, p = 0.008; Figure 2a) and total amount (F_1,33_ = 8.384, p = 0.007). The experimental manipulation of the diet had no significant effect on the deposition of γ-tocopherol, either in terms of concentration (F_1,33_ = 0.22, p = 0.65; Figure 2b) or in terms of total amount (F_1,33_ = 0.30, p = 0.59). There was a negative effect of the high quality diet on the concentrations of yolk carotenoids (F_1,33_ = 6.86, p = 0.01; Figure 2c) and a tendency for a similar effect on the total amount of yolk carotenoids (F_1,33_ = 3.31, p = 0.08).

The diet had a significant effect on the composition of yolk carotenoids and α/γ-tocopherol. There was a positive correlation between yolk α-tocopherol and carotenoids under the more “natural” conditions of the low quality diet (Pearson’s r = 0.66, p = 0.002), but not in the high quality group (Pearson’s r = −0.22p = 0.41). The correlations were in fact significantly different (z = −2.72, p = 0.007). By contrast the relationship with γ-tocopherol did not vary significantly with diet (γ-tocopherol and carotenoids, HQ: Pearson’s r = 0.41, p = 0.12; LQ: Pearson’s r = 0.48, p = 0.04; γ-tocopherol and α-tocopherol, HQ: Pearson’s r = 0.32, p = 0.18; LQ: Pearson’s r = −0.07, p = 0.81).

Interestingly, the total amount of yolk carotenoids and γ-tocopherol increased with yolk mass (carotenoids: Pearson’s r = 0.61, p = 0.0001; γ-tocopherol: Pearson’s r = 0.49, p = 0.003), but the concentrations of yolk carotenoids and γ-tocopherol were independent of yolk mass (carotenoids: Pearson’s r = 0.03, p = 0.85; γ-tocopherol: Pearson’s r = 0.14, p = 0.41). Yet, this was not the case for α- tocopherol, neither the total amount (Pearson’s r = 0.21, p = 0.22) nor the concentrations varied significantly with yolk mass (Pearson’s r = −0.14, p = 0.44).

We also studied the covariation of yolk carotenoids and α/γ-tocopherol with the yolk testosterone concentrations, given that their deposition may be mutually adjusted [21] (the yolk testosterone concentrations for all eggs were measured as separate part of the study, for all details see [40]). There were no significant relationships between the carotenoid or α/γ-tocopherol levels, respectively, and the concentrations or total amount of yolk testosterone (concentrations: Pearson’s r: -0.13 ≤ r ≤ 0.23, 0.11 ≤ p ≤ 0.78; content: Pearson’s r:-0.17 ≤ r ≤ 0.21, 0.23 ≤ p ≤ 0.87). Interestingly, the concentrations of yolk testosterone (Pearson’s r = −0.15, p = 0.007), but not the total amount of testosterone (Pearson’s r = −0.02, p = 0.89) decreased with increasing yolk mass.

### Trans-generational effects: consequences of the early growth conditions

**Figure 3:**
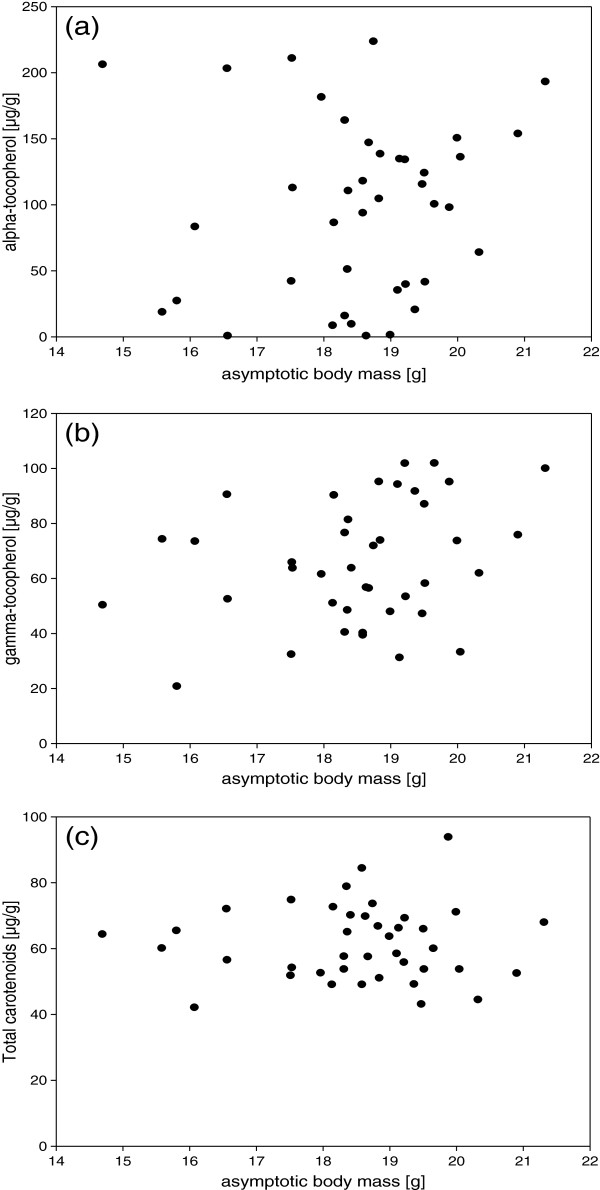
**Long-lasting consequences of past environmental effects that acted during the early developmental period.** Relationship between the asymptotic body mass (indicator of the early developmental conditions) and the concentrations of alpha-tocopherol (**a**), gamma-tocopherol (**b**) and carotenoids (**c**) in the yolk of their eggs laid as adult. In neither case was the relationship statistically significant.

Individual growth trajectories were determined for all daughters that were part of the inheritance experiment (N = 40). Yet, there was no clear evidence for a significant negative effect of harsh conditions during growth as indicated by a low asymptotic body mass (A), on yolk α-tocopherol [Pearson’s correlations, concentration: r = 0.09, p = 0.58 (Figure 3a), total amount: r = 0.07, p = 0.67], yolk γ-tocopherol [Pearson’s correlations, concentration: r = 0.28, p = 0.08 (Figure 3b), total amount: r = 0.22, p = 0.18] or yolk carotenoid [Pearson’s correlations, concentration: r = 0.2, p = 0.91 (Figure 3c), total amount: r = −0.04, p = 0.80] deposition. Neither was there a significant relationship between growth rate (k) and yolk α-tocopherol [Pearson’s correlations, concentration: r = 0.24, p = 0.14, total amount: r = 0.28, p = 0.08], yolk γ-tocopherol [Pearson’s correlations, concentration: r = 0.14, p = 0.38, total amount: r = 0.18, p = 0.27] or yolk carotenoid [Pearson’s correlations, concentration: r = 0.19, p = 0.23, total amount: r = 0.24, p = 0.13] deposition.

## Discussion

Maternal effects occur when the phenotype of the offspring is influenced by the phenotype of the mother [1,2]. The phenotype of the mother in turn depends on her genetic make-up, on the current environmental conditions acting on her, as well as on the environmental conditions that affected her in the past, such as during early development, when the environment can have profound effects on adult phenotype [33]. All of these pathways may form significant sources of variation in maternal effects. Here, we focused on the maternal transfer of carotenoids and α/γ-tocopherol to the yolk, which are maternal traits generating significant phenotypic variation in offspring [18,41]. We studied all three above mentioned sources of variation, of which neither the contributions of past environmental conditions nor genetic effects have been studied before. The different sources of variation will be discussed successively, and we will pay particular attention to potential underlying physiological mechanisms.

### Genetic effects: individual consistency and inheritance

Our study is to the best of our knowledge the first to report on genetic components of yolk carotenoid and vitamin E deposition, despite the importance of determining the heritability of traits for our understanding of evolutionary processes. A number of physiological processes (absorption, transportation, metabolism and deposition) are involved in the transfer of carotenoids and vitamins to the ova/yolk [28,29,42], and any of these mechanisms may be subject to heritable influences. However, we found no evidence for significant intra-individual repeatability in the transfer of carotenoids and α/γ-tocopherol to the yolk, and no significant evidence for heritable variation. Instead, females were highly inconsistent in their transfer of carotenoids and α/γ-tocopherol to the yolk, even though experiencing very similar environmental conditions, with exception of their mate of which we do not have any quality-related information. However, the potential effect of mate quality on yolk antioxidant deposition is as yet unclear (no evidence: [43]; positive evidence: [44]). Large among year variation has also been reported in the wild, but these studies did not analyze their data at the level of the individual [8,24]. Yet, our estimate of individual consistency is probably very conservative, as we exclude pseudo-repeatability as may be found in the wild where females may return to their previous nest site and mate with the same partner.

The small and non-significant mother-daughter resemblance fits with the lack of individual consistency, indicating a low heritability. However, these results have to be interpreted cautiously given the sample size (but see also [13,14]). The data nevertheless suggest that the transfer of carotenoids and vitamin E may represent an example where the environmental variation passes largely unmodified through the mother into the offspring (see [45] and references therein). The large among population variation in yolk carotenoid concentrations as has been reported in a previous study has consequently been ascribed to be resource-dependent rather than reflecting genetic local adaptation [46]. However, again our results have to be interpreted carefully, given the negative heritability estimate for α-tocopherol, which indicates a low reliability a common problem to single parent-offspring regressions [32].

Differences in the mechanisms available to the female, in particular the passive nature of the transport of carotenoids and vitamin E via the bloodstream (see above), may explain why the heritabilities reported here are lower than those for yolk testosterone [13-15]. This topic clearly deserves further exploration. However, the fact that this study is the first to describe the lack of heritable variation in the transfer of carotenoids and α/γ-tocopherol currently limits the possibilities for further comparison. In dairy cattle, the heritable component for carotenoid concentration in milk was low (h^2^ = 0.11 ± 0.10), while it was much higher for the blood carotenoid concentrations (h^2^ = 0.46 ± 0.16) [47]. In birds, the variance in plasma carotenoid levels of nestlings was largely explained by current environmental conditions, indicating a very low degree of genetic inheritance [48-50]. Yet, these studies were performed during the period of parental care, which likely reinforces common environment effects. Significant genetic variation in the incorporation of carotenoids into flesh has been found in sockeye salmon *Oncorhynchus nerka*[42], and there is some evidence that the carotenoid pigmentation of integument and yolk are associated in birds [11].

These traits discussed above are certainly to some extent (mechanistically) different from the deposition of carotenoids and vitamin E to the yolk. However, they may share some of the same physiological processes such as absorption in the gut, transportation in the bloodstream and metabolism, and as such most studies so far point towards a low level of heritable variation, which is in line with our study. The low mother-daughter resemblance also indicates that maternal effects acting prior to birth do not prime the carotenoid and vitamin E metabolism such that the transfer to the yolk is affected, contrasting previous evidence for the importance of prenatal maternal effects for carotenoid-based coloration (beak color: [18]; plumage coloration: [38]). The effects of yolk carotenoids on the subsequent capacity of the chick to assimilate dietary carotenoids [51] may thus be temporarily limited or trait-specific.

### Environmental effects - the current environment

The diet [seed only (LQ) versus seed and supplements (HQ)] prior to and during laying had a significant effect on the carotenoid and vitamin E composition of the yolk. Concentrations as well as total amounts of yolk α-tocopherol increased in the HQ diet, which is most likely a result of the Vitamin E enriched egg food. 75% of the vitamin E in the yolk measured here was α-tocopherol, which has the highest antioxidant potential [52]. There was no change in γ-tocopherols, but γ-tocopherol is the predominant form of vitamin E in seeds [53], suggesting that it may not have been limited in either of the diets. The concentrations of carotenoids, however, were lower in the HQ group. Carotenoids were neither incorporated in the egg food, nor were they enriched in the other supplements of the HQ diet. Yet, females on the HQ diet may have preferentially fed on the supplements in particular the protein-rich egg food, which were, however, poor sources of carotenoids.

Thus, our results underline the importance of the availability of carotenoids and vitamins in the predominant diet during egg formation in determining their concentrations in the yolk [8-11,45,54-56]. Yet, diet-dependent changes in yolk carotenoid and α/γ-tocopherol composition as observed in this study not only reflect availability and individual food preferences, but may also relate to physiological processes such as competitive exclusion and selective absorption in the uptake of nutrients in the gut [57].

Interestingly, the regulation of transfer of different antioxidant components appeared to be independent of each other, in that the relationship of carotenoids and α/γ-tocopherol varied with diet. Yolk α-tocopherol and carotenoids levels correlated under LQ/standard conditions, but not in the protein enriched HQ group. This suggests that a correlation between levels of carotenoids and vitamin E in egg yolk as often found in previous studies [21,43,58], may be due to a correlated availability in dietary sources.

The total amount but not the concentrations of both carotenoids and γ-tocopherol increased with yolk mass as found in a previous study [59] (see also [43]). This indicates that their deposition may be coupled to the yolking process, e.g. in terms of a passive transfer along with proteins and lipids. In contrast, when analyzing the relationship between yolk mass and yolk testosterone, the pattern appeared to be the opposite: the concentrations but not the total amount of testosterone decreased with increasing yolk mass. This suggests a mechanism of transfer that is rather independent of yolking, which is in line with the fact that the predominant amount of yolk testosterone is secreted from specific cells surrounding the oocyte [26]. On the other hand carotenoids and vitamins are transported to the yolk via the blood stream and lipoprotein carriers [60,61]. The levels of antioxidants and testosterone were not significantly correlated [43,62] (but see [9,44]), but a correlation between both may, given the different pathways, only be expected if a common environmental factor influences both traits simultaneously or to a similar extent. Further insight into the mechanisms and the potential role of depletion may be gained from studying within-clutch patterns (e.g. [59,62]). However, this cannot be further addressed here, as we did not investigate the within-clutch variation.

However, neither the total amount nor the concentrations of α-tocopherol varied with yolk mass. At present we can only speculate about the causes for this, such as their specific ability to bind to different classes of lipoproteins with different polarity in the blood stream, and these different types of lipoproteins may vary with the diet [61] or a super-abundance of α-tocopherol and a saturation of one of the mechanisms involved in their deposition. However, the latter contrasts with a previous study showing that larger yolks contained more carotenoids in a year with high carotenoid availability, but not in a year with lower availability of carotenoids [24].

### Environmental effects – in the past

The consequences of past environmental conditions were estimated on the basis of individual early developmental growth trajectories. We calculated the asymptotic mass and the rate of growth for each individual of the F1 generation. Both measures can serve as an indicator for how well a chick was growing during the first 20 days of life, which again reflects the environmental conditions it experienced. However, we did not find strong evidence that the transfer of carotenoids or vitamin E to the yolk was affected by the growth conditions experienced during the nestling period. We hypothesized that the latter would be the case since two recent studies have shown that early developmental conditions impinge on the expression of carotenoid-dependent traits at adulthood (beak color: [36]; plumage coloration: [37,38]). Based on these studies we expected that the early developmental conditions may affect the carotenoid metabolism at adulthood – with potential consequences for the transfer of carotenoids to the yolk. But our data do not support this idea, and an alternative explanation for the observed pattern may be an effect of developmental stress on structural components of the feather or beak. Yet, it has to be kept in mind that our data are correlative and may as such also reflect intrinsic (genetic) difference as the growth conditions were not experimentally manipulated in this study. However, we did find an effect of the early developmental conditions on yolk testosterone levels [14], which argues against the possibility that the conditions were too mild in order to exert a measurable effect.

## Conclusion

The aim of this study was to unravel the contributions of genetic and environmental effects on the maternal transfer of carotenoids and vitamin E, while also paying attention to the underlying physiology. The availability of carotenoids and vitamin E in the diet was the only component investigated that was significantly related to the yolk antioxidant composition. The transfer of yolk carotenoids and to some extent vitamin E appeared to be coupled to the process of yolk formation, representing another pathway along which diet and food conditions may alter the yolk antioxidant composition. Our results show a strong influence of the current but not the past environment, and traits that are influenced heavily by environmental factors typically show low heritabilities [32,63].

Using a captive (bird) species, allows studying traits that are difficult to measure in the wild due to e.g. low recruitment rates (reviewed in [42]), and provides meaningful estimates of heritability (reviewed in [46]). However, while controlling for potentially confounding factors that complicate studies in the wild may be beneficial in some circumstances, other questions can be better answered using study systems in the wild. For example, otherwise limiting resources become equally available in captivity, which [64] may in turn diminish (intrinsic) differences between individuals. The latter has to be taken into account when interpreting the results as obtained in captivity as biological meaningful insights into natural systems [65].

## Materials and methods

### Individual consistency, inheritance and early developmental effects

This study was conducted using two generations of *Fife Fancy* canaries. All animals were handled in accordance with good animal practice and the experiments have been conducted according to Belgian legislation for animal experimentation approved by the Ethical committee of the University of Antwerp (permit number 2006/19). The birds of the first generation were obtained from local breeders or originated from our own breeding program. In the first year, all females that were assigned to the *parental generation* (=P-generation) laid two clutches: the first clutch was initiated in March, five weeks after the light regime was changed to 14:10 L:D (breeding conditions). This clutch was collected for analysis and to this end replaced by dummy eggs. We weighed the eggs (to the nearest 0.01 g), and then froze them all at −18°C. Only the second-laid egg was subsequently used for the analysis of yolk carotenoid and α/γ-tocopherol (vitamin E). All dummy eggs were removed two days after clutch completion, the pair was divided, and returned to large stock cages (separated for sex). In mid-May, all females of the P-generation were mated with a new partner, by then housed under natural light–dark conditions. Only their second clutch was allowed to hatch (producing the F1 generation). Chicks were marked at hatching with a non-toxic pen and their sex was determined molecularly using a droplet of blood at hatching [66]. The following day (if possible), all female chicks were selected for the experiment, weighed (to the nearest 0.01 g), and cross-fostered, creating female-only broods of four chicks of similar weight. As such we control for potentially confounding effects of brood sex ratio, brood size and hatching order. Male chicks were used in a different experiment [67]. Body mass gain (to the nearest 0.01 g) of all chicks was measured early in the morning until day 20, when the growth curve has levelled off. Based on the body mass measures we calculated for each individual its asymptotic body mass and its growth rate (see: *Statistical analyses*). We separated the chicks from their parents at independence (about 30 days old). All birds were subsequently kept in large single-sex aviaries till the next year.

In the subsequent year, all (remaining) females of the P-generation that sired a daughter in their second clutch of the previous year, as well as (one of) their daughter(s) (F1) were allowed to breed in March, five weeks after the light regime was changed to 14:10 L:D. Females of the P-generation were mated with a male they had not been mated with before and females of the F1 with an unrelated partner. All clutches were collected again for analysis according to the previously described procedure. All males used in this experiment were unrelated to their partner, only used once, one-year old and randomly assigned.

Throughout the experiments, we kept the pairs in separate breeding cages equipped with nest boxes and nesting material. We provided the birds with canary seed mixture (van Camp, Belgium), water, shell grit, and cuttlefish bone ad libitum and twice weekly (after the chicks hatched daily) with egg food (van Camp, Belgium). During laying, nests were checked daily and the eggs were marked in order to keep track of laying date and laying position.

### Environmental effects (food manipulation)

A different set of females was randomly assigned to a low quality (20 g of standard canary seed mixture each week, equals the average individual seed consumption; N = 22) or high quality diet [standard canary seed mixture ad libitum (allowing selective feeding), 10 g of egg food (high protein content, which incorporated vitamin E; Van Camp, Belgium) and apple, carrot, or germinated seeds, respectively, every other day; N = 22] group for the entire duration of the experiment (9 weeks) (for more details see also [40]). Females were randomly mated with an unrelated male, after five weeks of food manipulation (for similar protocols see: [68,69]). The breeding pair received the same diet that the female was assigned beforehand, but adjusted for the number of birds in the cage (=40 g seeds in case of the low quality group). The pairs were provided with nest boxes and nesting materials. The nests were checked daily for egg laying. After being replaced by a dummy egg, freshly laid eggs were weighed and immediately stored at −18°C.

### Yolk carotenoid and vitamin E (Î±/Î³-tocopherol) analysis

We standardized our analysis by selecting eggs of the same laying position (the second-laid egg) for biochemical analyses, since yolk carotenoid and α/γ-tocopherol may vary systematically with laying order [8,9,11,59]. Typically, levels of egg components are highly correlated within clutches (for yolk androgens see [46,70], for yolk antibodies see [71]), and the systematic within clutch variation in yolk carotenoids and α/γ-tocopherol [8,9,11,59] suggests that this also applies for yolk carotenoids and α/γ-tocopherol. Partial sampling of a clutch via single eggs is thus likely to provide a reliable estimate of the clutch level (see [7,13,14,46] for a similar approach). Samples (0.5 ml) of pre-diluted yolk (1:1 w/v in water, [40]) were thawed and added to 0.5 ml of 5% sodium chloride and mixed by vortexing. Next, 1 ml ethanol was added and samples were homogenised for 20 s, then 2 ml hexane was added and the mixture was homogenised for a further 20 s. After centrifugation the hexane phase containing the carotenoids and vitamin E was collected. The extraction step using hexane was performed once more and the extracts were combined. Total carotenoids were determined using absorbance spectrophotometry at 450 nm (Nicolet Evolution 500), and concentrations calculated using the extinction coefficient of lutein in hexane (2589; [72]). Hexane (1 ml) was then evaporated to dryness under a stream of N_2_, then the residue was redissolved in 150 μl dichloromethane and 150 μl methanol ready for HPLC. For analysis of vitamin and E (γ- and α-tocopherol), samples (10 μl) were injected into an HPLC system fitted with a Spherisorb type S30DS2, 3 μ C_18_ reverse-phase column (15 cm x 4.6 mm) (Phase Separations, Clwyd, UK), and a mobile phase of methanol-distilled water (97:3) at a flow rate of 1.05 ml min^-1^. Fluorescence detection of tocopherols involved excitation at 295 nm and emission at 330 nm. Standard solutions of tocopherols (Sigma-Aldrich, Poole, UK) in methanol were used for quantification.

### Statistical analyses

We collected eggs for 40 mothers (P-generation), of which 25 females were sampled in both years, and also collected eggs of their daughters (F1) (one female from the P generation was excluded from the heritability estimation as the respective second-laid egg broke before freezing/subsequent measurements). We calculated the consistency in yolk carotenoids and vitamin E concentration and their total amount using the second-laid egg as r = s^2^_among females_/(s^2^_within females_+ s^2^_among females_) [73]. The consistency is thought to provide an upper-bound estimate for the heritability of a given trait [32]. We then calculated the heritability of all traits of interest as twice the slope of the respective mother-daughter regression [32]. For all analyses we used mean values for the P-generation, if a female was sampled in both years.

We fitted logistic growth curves based on the body mass measurements between day 1–20 by least squares regression (SPSS 14.0) using the model: W = A/(1 + e^-k*(t-ti)^), in which W is body mass at a given age t [d], A [g] is the asymptotic body mass that the nestling approaches, k [d^-1^ is the logistic growth constant, reflecting the rate at which the mass increases from initial mass to asymptotic mass, and t_i_ is the point of inflection when growth changes from accelerating to decelerating [d] [73]. These estimates represent established, biologically interpretable measures of the individual growth trajectory and thus the quality of the early development [55,67,74-76]. We hence used the asymptotic mass and the logistic growth constant in order to estimate the consequence of the early developmental conditions on yolk carotenoid and vitamin E deposition at adulthood. Individuals will likely differ in their growth despite ad libitum food conditions, which is thought to relate to variation in parental food provisioning and within-brood size asymmetries [77]. However, it has to be taken into account, given the correlative nature of the data, that such variation may also relate to intrinsic genetic variation in growth.

The covariation among egg components and egg traits were investigated via Pearson’s correlations. The effects of diet on the different egg traits were investigated by means of analyses of variance with diet as a fixed factor. All analyses were conducted in SPSS 14.0 and data are shown as mean ± se unless stated otherwise.

## Competing interests

The authors declare that they have no competing interests.

## Authors’ contribution

WM designed the experiment, was involved in data collection, analysed the data and drafted the manuscript, JV collected data and participated in design and data analysis, ME and JDB contributed to the analysis, interpretation of the data and manuscript preparation. All authors read and approved the manuscript.
